# Case Report: Two cases of survival after complete transection of the left common carotid artery

**DOI:** 10.3389/fsurg.2022.1082658

**Published:** 2023-02-03

**Authors:** Xin Yang, Zhonglu Liu, Qi Sun, Yakui Mou, Chuanliang Jia, Wenbin Zhang, Fei Song, Wen Li, Hua Zhang, Xicheng Song

**Affiliations:** ^1^ Department of Otorhinolaryngology Head and Neck Surgery, Yantai Yuhuangding Hospital, Qingdao University, Yantai, China; ^2^ Shandong Provincial Clinical Research Center for Otorhinolaryngologic Diseases, Yantai, China; ^3^ Department of General Surgery, Yantai Municipal Laiyang Central Hospital, Laiyang, China

**Keywords:** complete common carotid artery transection, trauma, survival, left common carotid artery, CTA complete common carotid artery transection, CTA

## Abstract

Penetrating carotid artery traumas are rare yet fatal injuries with a high rate of mortality, and survivors may live with neurological sequelae. Of all the types of penetrating carotid artery traumas, the total transection of the common carotid artery (CCA) may be the most serious, can lead to death quickly, and has few reports of survivors. We described two cases of patients with complete CCA transections who survived without any neurological sequelae. The penetrating neck traumas of both patients were confirmed as complete CCA severance by CT and surgical exploration. Case 1 received the insertion of an interposition polytetrafluoroethylene graft to reconstruct the CCA, with postoperative ultrasound and CT angiography (CTA) verifying the total occlusion. Case 2 underwent nonoperative management under close observation and did not develop delayed active bleeding or neurological symptoms. Both patients recovered well, and no nervous system sequelae appeared during the follow-up period. A carotid artery injury cannot be ruled out in an asymptomatic penetrating neck injury. If CTA is feasible given the patient's hemodynamic condition, then it should be used as a routine examination to evaluate cervical vascular injury in patients with penetrating neck trauma. Management for hemodynamically stable carotid artery injuries remains controversial. These two cases of transverse carotid artery injury have caused us to further consider the principles of this kind of case management.

## Introduction

Penetrating carotid artery injuries are uncommon yet fatal lesions, leading to death through massive hemorrhage or survival with neurological sequelae. Among these injuries, the complete common carotid artery transection (CCCAT) may be the most severe, can lead to fatal bleeding quickly, and has an extremely low survival rate. The cases of two patients with CCCAT who survived and had no neurological symptoms during the follow-up of more than two years are explored below.

## Case presentation

### Case 1

**Figure 1:**
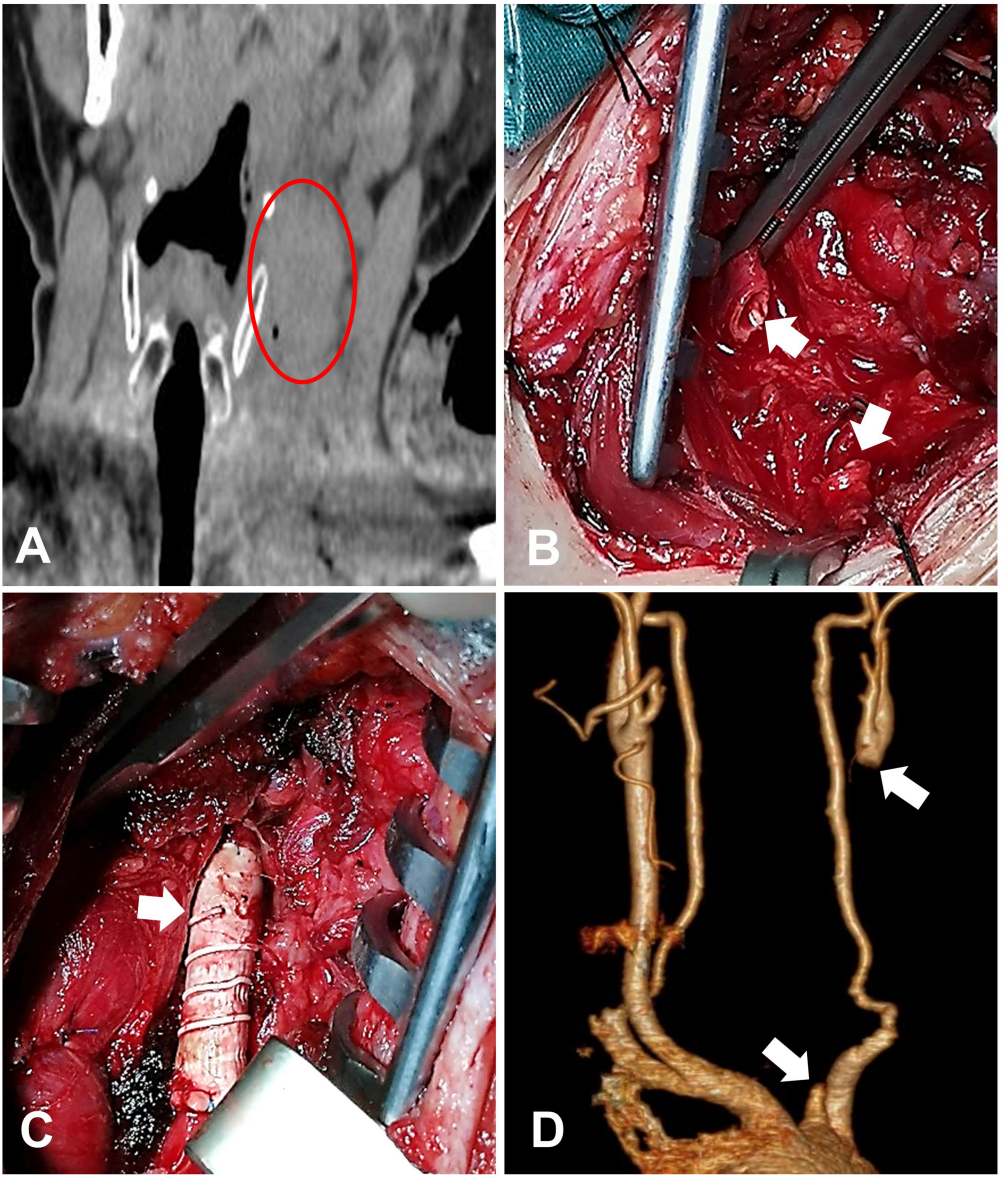
Case 1: (**A**) coronal CT of the neck showed massive blood clots around the left carotid artery, which compressed and displaced the trachea. Note: the red circle is marked in the left carotid sheath. (**B**) Intraoperative exploration of left common carotid artery amputation. Note: the arrow is marked as the broken end of the left common carotid artery. (**C**) Place the image of an artificial vascular anastomosis. Note: the arrow is marked after artificial vascular implantation. (**D**) Three-dimensional postoperative computer tomographic scan showing a clogged left carotid. Note: the arrow points to the broken end of the left common carotid artery.

The patient, a 40-year-old male, was admitted to the hospital 2 h after being stabbed in the left neck. The vital signs on admission were as follows: body temperature 35.9°C; heart rate 98 beats/min; blood pressure 100 mmHg; respiratory rate 19/min; and fingertip oxygen saturation 100%. An open wound of approximately 4.0 cm and ongoing bleeding were found in the left supraclavicular fossa. The amount of bleeding was approximately 500 ml. No evidence of respiratory distress was noted. The patient was estimated to be clinically stable given his sober consciousness, normal physical movement, cooperation in physical examination, and hemodynamic stability. CT scan (Figure 1A) indicated soft tissue swelling, gas accumulation in the neck, and massive blood clots around the left carotid artery which compressed and displaced the trachea. The patient underwent operative exploration immediately. We asked the vascular surgeon to consult and handle the vascular injury. During the operation, the clots were removed, followed by active bleeding mainly from the ruptured jugular vein which was then repaired with 5-0 polyethylene thread. Carotid artery injury was suspected because the pulsation of the internal carotid artery (ICA) was not palpable. Further surgical exploration revealed that the left common carotid artery (CCA) was completely transected at 4 cm below the bifurcation of the CCA, and the irregular proximal and distal ends were filled with thrombus. Then, we applied a vascular clamp to the broken end of the blood vessel to remove the thrombus in the proximal and distal vessels. Blood flow was unobstructed after releasing the hemostatic forceps. After an approximately 1-cm resection of the irregular stumps, end-to-end anastomosis was no longer possible (Figure 1B). Thus, the insertion of an interposition polytetrafluoroethylene (PTFE) graft (Figure 1C) was performed to reconstruct the CCA, and the patient was transferred to the ICU after the operation. Anticoagulant agents and antibiotics were given to improve the long-term patency of the reconstructed CCA and prevent infection. However, on the 8th day after the operation, ultrasonic examination showed thrombosis in the distal segment above the PTFE graft, and the flow velocity of the left ICA decreased. Consultation recommendations included vascular surgery. As the patient showed no neurological symptoms, no further intervention was performed. Two weeks after discharge, CT angiography (CTA) showed that blood flow was absent at the site of the PTFE graft but present in the internal and external carotid arteries above the bifurcation, these arteries being thinner than their counterparts on the opposite side (Figure 1D). The patient showed no physical and neurological sequelae during the follow-up by telephone for more than 2 years after discharge.

### Case 2

**Figure 2:**
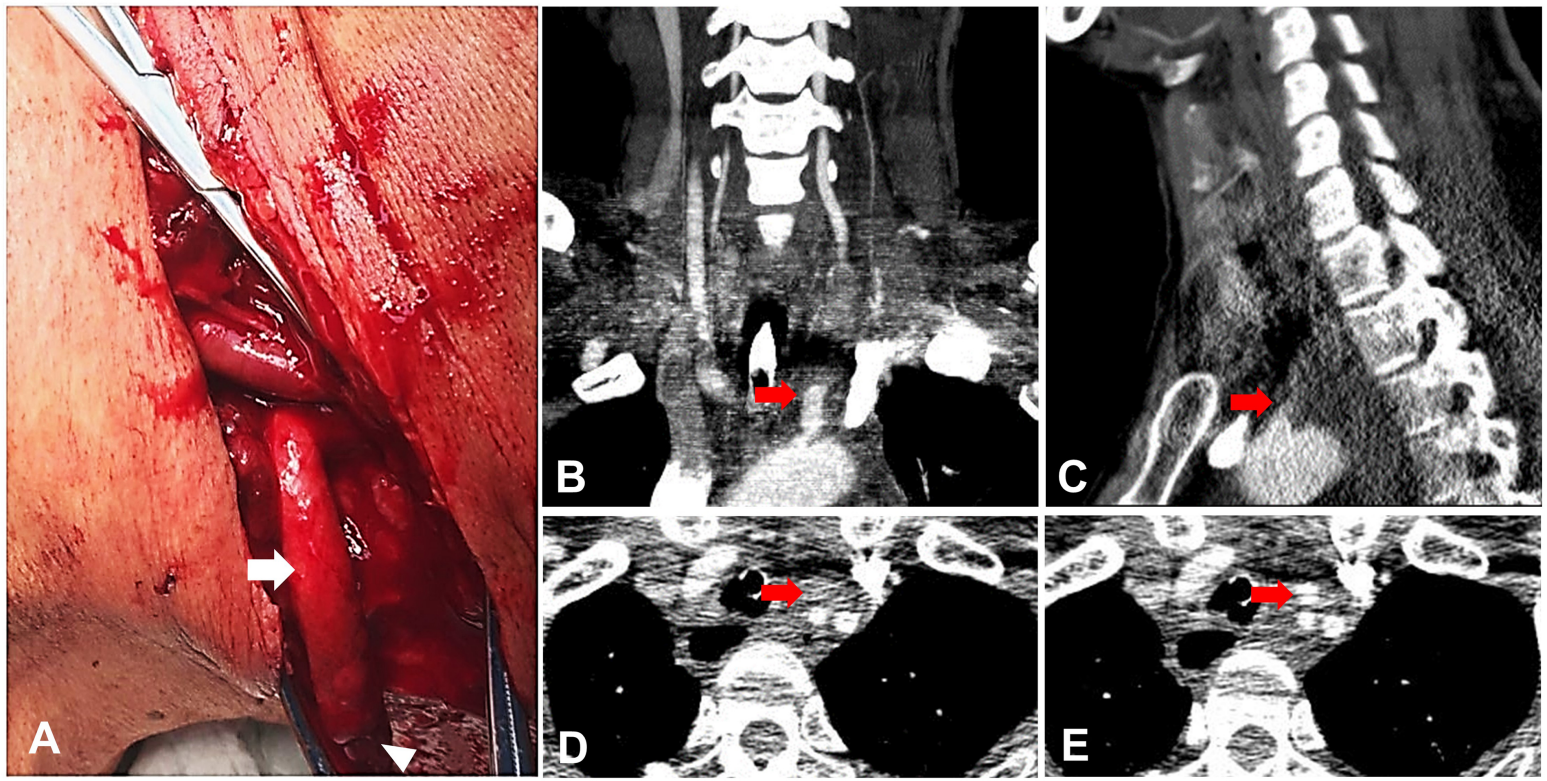
Case 2: (**A**) the left common carotid artery that was severed from the proximal end of the heart and prolapsed at the time of treatment. Note: the arrow points to the left common carotid artery. The triangle points to the distal end of the left common carotid artery. The cervical CT coronal (**B**) and sagittal (**C**) images showed that the origin of the left common carotid artery was not shown, the contrast filling was not seen in the distance, and the structure was not clearly displayed. Note: the arrow refers to the beginning of the left common carotid artery. (**D**) The left common carotid artery was not seen at the CT level of the neck. Note: the arrow refers to the location of the left common carotid artery. (**E**) Normal development of the left common carotid artery at the level of the CT in the neck. Note: the arrow refers to the left common carotid artery.

The patient, a 51-year-old male who suffered from depression for more than 3 years, was admitted to the local hospital 2 h after he attempted suicide by cutting his neck. The patient was conscious, with ongoing bleeding in the neck, and a blood pressure of 70/40 mmHg on admission. He underwent initial management including massive transfusion, pressor agent therapy, and operative exploration. During the operation, the left CCA was identified to be completely transected with approximately 6 cm of the distal end exposed on the wound surface (Figure 2A). Exploration of the origin of the CCA was not performed because of potential life-threating hemorrhage. After repairing the damaged internal jugular vein and ligating the superficial vein including the external jugular vein, the distal end of the CCA was also ligated to stop the reflux bleeding after the blood pressure increased to normal. After ensuring that no active bleeding was present, the severed cervical muscles and the incision were sutured, leaving the undetected proximal end of the CCA untreated because of the difficulty of exposure and potential risk of uncontrollable massive hemorrhage. Then, the patient was transferred to our hospital ICU under sedation and with neck bandaging, endotracheal intubation, and pressor drugs to maintain his blood pressure. On admission, vital signs were as follows: body temperature 36.0°C; heart rate 75 beats per minute; blood pressure 95/61 mmHg; respiratory rate 12/min; and fingertip oxygen saturation 100%. Contrast CT scan further identified the CCCAT according to the proximal stump of the CCA and the absence of the prograde blood flow through the CCA (Figures 2B–E). Then, a multidisciplinary consultation was held with the participation of the Ear, Nose, and Throat (ENT), vascular surgery, and cardiothoracic surgery departments. Cardiothoracic surgery suggested thoracotomy, and vascular surgery suggested occlusion with coils. However, thoracotomy entailed high risk, trauma, and high cost. By contrast, coil implantation entails the risk of re-bleeding. The vital signs of the patient were stable at that time, no obvious active bleeding occurred, comprehensive evaluation of the patient's condition was conducted, and finally the patient and his family chose conservative treatment. The patient was kept in careful observation for 1 week in the ICU before discharge, and no bleeding and neurological symptoms appeared. He was in good condition and remained neurologically asymptomatic during the follow-up by telephone for more than 2 years after discharge.

## Discussion

Carotid artery injury accounts for approximately 6% of penetrating neck injuries (1) and is described as trauma to the neck that has breached the platysma muscle (2), causing death through massive hemorrhage or survival with an irreversible neurological deficit. In the two cases reported in this paper, the injuries involved complete transection of the left CCA for a significant duration of time prior to arrival at the hospital without fatal hemorrhage or neurological deficit, and no neurological symptoms appeared during the follow-up period after discharge. No similar cases were reported. Andrási et al. reported a survival after suicidal transection of the CCA (3), which occurred in the hospital where rescue measures, such as local pressure of the wound, transfusion, and clamping of both arterial stumps, were implemented quickly, a situation which obviously differs from the cases we reported. O’Banion et al. performed a cohort study of 50 patients (CCA: 3; ICA: 47) with traumatic carotid injuries who were initially managed nonoperatively: only one patient ultimately required conversion to surgery (4). However, injury types, such as occlusion, transection, partial transection, and pseudoaneurysm, were not provided by O’Banion et al., nor did they provide the imageological and pathological details of the injured arteries, details that are vital for analyzing the mechanism of temporary or permanent hemostasis of the injured major carotid arteries.

Both CCA and ICA injuries carry a high risk of death through massive hemorrhage and neurological sequelae, and their treatment remains controversial. At present, no consensus exists on the management of injured arteries in patients with stable hemodynamics and without neurological dysfunction. Some scholars suggest reconstruction of the artery if technically possible (1, 5); others propose ligation or mere observation as the preferred treatment in such situation (6). In addition, some researchers present views that fall somewhere between the two above approaches, proposing nonoperative management as acceptable in well-selected patients (7). However, few independent reports are available on carotid artery transection. In the cases we reported, the patient in Case 1 underwent surgical exploration and the insertion of an interpositional PTFE graft for the CCA reconstruction. Postoperative CTA showed no prograde flow through the reconstructed CCA, indicating that the CCA was completely occluded. We postulate that this outcome may be related to anastomotic stenosis, the patient's hypercoagulable state, local inflammatory reaction, intimal injury, and subsequent thrombosis. Given unsuccessful restoration of vascular continuity and the ideal outcome of the patient without stroke or neurological sequelae, ligation of the injured CCA is also proposed as a feasible surgical option in such circumstances. The patient in Case 2 was transferred to our hospital with stable hemodynamics, without active bleeding or neurological symptoms, after repair of the internal jugular vein, ligation of the distal end of the transected CCA, and suturing of the incision in the local hospital, leaving the proximal end of the CCA untreated. Given the stable condition of the patient, the CT results revealed no prograde flow through the CCA. Note that the option of an open or endovascular surgery entails difficulty and may lead to considerable trauma and potentially life-threating massive bleeding. Finally, the patient's family chose nonoperative management of the transected artery. The mortality rate of penetrating neck trauma is estimated to be as high as 6%, with massive hemorrhage being the most common cause of death (8). Patients presenting with penetrating carotid trauma have an overall stroke rate of 17% (4), and more specific data about CCCAT is not available. In this paper, no fatal massive hemorrhage occurred in these two patients with CCCAT despite the long duration of time before the availability of hemorrhage control treatment, thereby suggesting that CCCAT may not necessarily cause death through massive hemorrhage. We postulate that the bleeding following the rupture of the carotid artery can stop spontaneously in some circumstances. Although the CCAs eventually failed to recanalize, neither patient experienced stroke or neurological symptoms, indicating that the choice between revascularization or ligation of the CCA should be carefully balanced in the treatment of CCCAT, especially in the presence of a complete Willis circle (CTA finding) and good distal regurgitation of the artery (operative finding). In addition, we learned the following lesson from Case 1: CTA can be chosen as the first-line imaging modality for diagnosing vascular injuries in hemodynamically stable patients with neck trauma.

In summary, we shared two cases of CCCAT and found some interesting and confusing phenomena that have not been reported previously in the literature. We remain uncertain about the mechanism of automatic hemostasis in these two CCCAT patients. To date, no relevant literature has examined this rare phenomenon.

## Data Availability

The original contributions presented in the study are included in the article/Supplementary Material, further inquiries can be directed to the corresponding authors.

## References

[1] Demetriades D Asensio JA Velmahos G Thal E. Complex problems in penetrating neck trauma. Surg Clin North Am. (1996) 76:661–83. 10.1016/s0039-6109(05)70475-8 8782468

[2] Sperry JL Moore EE Coimbra R Croce M Davis JW Karmy-Jones R Western Trauma Association critical decisions in trauma: penetrating neck trauma. J Trauma Acute Care Surg. (2013) 75:936–40. 10.1097/TA.0b013e31829e20e3 24256663

[3] Andrási TB Gemechu A Spielberger J Rohsbach U Vitolianos N Vahl CF. Survival after suicidal transsection of the left common carotid artery in octogenarian. Am Surg. (2011) 77:E50–2. 10.1177/000313481107700306 21375829

[4] O'Banion LA Dirks RC Siada SS Dubose JJ Inaba K Byerly S Risk factors for stroke in penetrating carotid trauma—an analysis from the PROOVIT registry. J Trauma Acute Care Surg. (2022) 92:717–22. 10.1097/ta.0000000000003519 34991129

[5] Fry RE Fry WJ. Extracranial carotid artery injuries. Surgery. (1980) 88:581–7. PMID: . 7423378

[6] Navsaria P Omoshoro-Jones J Nicol A. An analysis of 32 surgically managed penetrating carotid artery injuries. Eur J Vasc Endovasc Surg. (2002) 24:349–55. 10.1053/ejvs.2002.1736 12323179

[7] Serna JJ Ordoñez CA Parra MW Serna C Caicedo Y Rosero A Damage control in penetrating carotid artery trauma: changing a 100-year paradigm. Colomb Med. (2021) 52:e4054807. 10.25100/cm.v52i2.4807 PMC8634279 34908620

[8] Burgess CA Dale OT Almeyda R Corbridge RJ. An evidence based review of the assessment and management of penetrating neck trauma. Clin Otolaryngol. (2012) 37:44–52. 10.1111/j.1749-4486.2011.02422.x 22152036

